# Impacto da Síndrome Metabólica Relacionada à Infecção por Helicobacter pylori Ativa na Hipertensão Arterial Sistêmica

**DOI:** 10.36660/abc.20210931

**Published:** 2022-08-25

**Authors:** Jannis Kountouras, Apostolis Papaefthymiou, Stergios A. Polyzos, Evangelos Kazakos, Elisabeth Vardaka, Maria Touloumtzi, Maria Tzitiridou-Chatzopoulou, Christos Liatsos, Ioanna-Konstantina Sgantzou, Jürg Knuchel, Michael Doulberis

**Affiliations:** 1 School of Medicine Ippokration Hospital Aristotle University of Thessaloniki Macedônia Grécia Second Medical Clinic, School of Medicine, Ippokration Hospital, Aristotle University of Thessaloniki, Macedônia – Grécia; 2 Department of Gastroenterology University Hospital of Larisa Larisa Grécia Department of Gastroenterology, University Hospital of Larisa, Larisa – Grécia; 3 First Laboratory of Pharmacology School of Medicine Aristotle University of Thessaloniki Macedônia Grécia First Laboratory of Pharmacology, School of Medicine, Aristotle University of Thessaloniki, Macedônia – Grécia; 4 Department of Nutritional Sciences and Dietetics School of Health Sciences International Hellenic University Macedônia Grécia Department of Nutritional Sciences and Dietetics, School of Health Sciences, International Hellenic University, Macedônia – Grécia; 5 School of Healthcare Sciences Midwifery Department University of West Macedonia Macedônia Grécia School of Healthcare Sciences, Midwifery Department, University of West Macedonia, Macedônia – Grécia; 6 Department of Gastroenterology General Military Hospital of Athens Atenas Grécia Department of Gastroenterology - General Military Hospital of Athens, Atenas – Grécia; 7 Radiology Department University Hospital of Larisa Larisa Grécia Radiology Department, University Hospital of Larisa, Larisa – Grécia; 8 Division of Gastroenterology and Hepatology Medical University Department Aarau Suíça Division of Gastroenterology and Hepatology, Medical University Department, Aarau – Suíça

**Keywords:** Helicobacter pylori, Hipertensão, Síndrome Metabólica, Aterosclerose, Dislipidemias

**Ao Editor**

Em sua metanálise, Huang et al.,^1^ concluíram que a infecção por Helicobacter pylori (infecção por H. pylori) está positivamente associada à hipertensão arterial sistêmica, particularmente pela introdução do teste respiratório diagnóstico de 13C-ureia, significando infecção atual por H. pylori.

Nesse sentido, a hipertensão arterial sistêmica é um dos parâmetros mais significativos da síndrome metabólica (SM), e sua patogênese pode consistir principalmente de uma interação nociva entre mecanismos vasculares, renais, neurais e hormonais, dos quais a ativação aumentada do sistema simpático sistema nervoso (SNS) e o sistema renina-angiotensina-aldosterona (SRAA) predominam^2^ A atividade aumentada do SNS é uma característica usual de hipertensão arterial sistêmica resistente, acompanhada de aumento da liberação de norepinefrina, significando um elemento neurogênico que contribui para o desenvolvimento da hipertensão arterial sistêmica; e a superativação do SNS está associada a morbidade e mortalidade de distúrbios cardiovasculares relacionados à SM.^3^ Além disso, a desregulação do SRAA, incluindo o SRAA sistêmico e cerebral, tem sido documentada como uma das principais causas de vários tipos de hipertensão arterial sistêmica; e a superativação do SRRA também contribui para a obesidade associada à SM e morbidade e mortalidade cardiovascular.^4^

Da mesma forma, a infecção por H. pylori também está associada a patologias sistêmicas relacionadas à SM, especialmente doenças cardiocerebrovasculares e neurodegenerativas, os desfechos da SM.^5 - 8^ Especificamente, a infecção por H. pylori parece contribuir para a resistência à insulina (RI), o principal mecanismo subjacente responsável pela SM,^9^ que também desempenha um papel crítico na patogênese e progressão das lesões de órgãos-alvo desencadeadas pela hipertensão arterial sistêmica.^10^ A SM contribui para um risco aumentado de desenvolver aterosclerose,^11^ e, nesse sentido, a invasão de H. pylori em ateroma foi detectada pela introdução da reação em cadeia da polimerase (PCR).^12^ Foi observada a colonização direta de H. pylori nas paredes arteriais foi observada. *H. pylori* está associada à rigidez arterial, marcador precoce de aterosclerose sistêmica correlacionado com hipertensão arterial sistêmica e preditor independente de complicações cardiovasculares e mortalidade por todas as causas. Assim, o H. pylori tem sido associado à aterosclerose relacionada à SM por meio de uma diversidade de mecanismos envolvidos, potencialmente desencadeando hipertensão arterial sistêmica. A infecção por H. pylori pode estar envolvida independentemente na aterosclerose e na hipertensão arterial por meio de mecanismos distintos das causas convencionais de aterosclerose, incluindo os três fatores de risco não convencionais para doença arterial coronariana homocisteína, fibrinogênio e lipoproteína(a).^6 , 13 , 14^

Além disso, a dislipidemia relacionada à SM está associada à hipertensão arterial sistêmica^15^ e, nesse sentido, a infecção crônica por H. pylori pode desencadear metabolismo lipídico anormal do hospedeiro, incluindo, além da lipoproteína(a) mencionada, o colesterol de lipoproteína de baixa densidade (LDL- C), colesterol de lipoproteína de alta densidade (HDL-C) e colesterol total (TC),^16^ também citados pelos autores;^1^ O HDL-C mais baixo relacionado ao H. pylori parece promover dislipidemia.^17^ Em contraste, a erradicação do H. pylori diminui significativamente os níveis de CT, TG, LDL-C e fibrinogênio, um fator de risco independente para doença cardiovascular,^6^ enquanto aumenta as concentrações de HDL-C.^18 , 19^ Além de dislipidemia e hipertensão arterial sistêmica, a erradicação do H. pylori também melhora outros parâmetros relacionados à SM, incluindo índice de massa corporal (IMC),^20^ RI,^21^ e status oxidante total.^22^ Portanto, a erradicação da infecção por H. pylori reduz a ocorrência de dislipidemia relacionada à SM e outros parâmetros, incluindo hipertensão arterial sistêmica,^23 , 24^ potencialmente prevenindo a ocorrência de doença cardiovascular relacionada à SM acompanhada de hipertensão arterial.

Dados recentes indicam que sarcopenia relacionada à SM, infecção por H. pylori, dislipidemia, hipertensão arterial sistêmica, diabetes mellitus, tabagismo, consumo de álcool e dieta (salgada e/ou condimentada) estão ligados a lesões pré-cancerosas da mucosa gástrica, incluindo atrofia gástrica, metaplasia intestinal e displasia.^25^ A este respeito, evidências recentes interessantes também indicam que pacientes bariátricos com infecção por H. pylori apresentam taxas basais significativamente altas das lesões pré-malignas gástricas mencionadas, incluindo atrofia gástrica e metaplasia intestinal acompanhada de RI e hipertensão arterial.^26^

Finalmente, a doença hepática gordurosa não alcoólica (DHGNA), recentemente renomeada como doença hepática gordurosa associada à disfunção metabólica (DHGM), é o componente hepático da SM também associada à infecção por H. pylori, que parece contribuir para o seu desenvolvimento e progressão;^27^ DHGNA/DHGM é associado a um risco aumentado de cerca de 1,6 vezes de desenvolver hipertensão arterial sistêmica; e características relacionadas à SM, incluindo IMC alto, dislipidemia e diabetes mellitus tipo 2 no contexto da infecção por H. pylori apresentam uma tendência maior para o desenvolvimento de DHGNA/DHGM. A respeito disso, dados recentes indicam que a infecção por H. pylori está relacionada com RI e aumento da permeabilidade intestinal, o que poderia contribuir para o desenvolvimento de DHGNA/DHGM;^28^ e a infecção ativa por H. pylori é independentemente positivamente associada com a gravidade da esteato-hepatite não alcoólica e fibrose, achados que sugerem prováveis implicações clínicas.^27^ Entre os pacientes com DHGNA/MAFLD, a prevalência de hipertensão arterial varia de 40-70%, e estudos relativos mostraram que DHGNA/MAFLD está fortemente relacionada ao risco aumentado de pré-hipertensão arterial sistêmica e hipertensão.^29^ Em contrapartida, além da redução da referida hipertensão arterial sistêmica, a erradicação do H. pylori aumenta particularmente o HDL-C e reduz o LDL-C,30 restaurando assim a atividade cardioprotetora da relação HDL-C/LDL-C e diminuindo o risco cardiovascular associado a DHGM.^30^

Observando os dados mencionados, a infecção por H. pylori parece apresentar efeitos pleiotrópicos além do trato gastrointestinal e evidências crescentes a associam à SM, incluindo hipertensão arterial sistêmica. Mais pesquisas são necessárias, no entanto, para esclarecer o impacto potencial do H. pylori relacionado à SM na hipertensão arterial sistêmica, que representa um grave problema de saúde pública com alta incidência e prevalência globais que continuam aumentando e podem contribuir para a alta morbidade e mortalidade global. Identificar H. pylori e DHGNA/DHGM relacionados à SM e outros distúrbios relativos – como importantes fatores de risco para hipertensão arterial sistêmica – pode ser útil para melhorar a predição de risco, identificar estratégias de prevenção primária e selecionar um programa terapêutico para hipertensão arterial sistêmica.
