# Supplementary material for: Impacto da Síndrome Metabólica Relacionada à Infecção por Helicobacter pylori Ativa na Hipertensão Arterial Sistêmica
Source: Arq Bras Cardiol. 2022 Aug 25;119(3):502–4. [Article in Portuguese] doi: 10.36660/abc.20210931 (PMC9438526; doi:10.36660/abc.20210931)
Supplement: Translation

# Translation: Impact of Active Helicobacter pylori Infection-related Metabolic Syndrome on Systemic Arterial Hypertension

**Authors:** Jannis Kountouras, Apostolis Papaefthymiou, Stergios A. Polyzos, Evangelos Kazakos, Elisabeth Vardaka, Maria Touloumtzi, Maria Tzitiridou-Chatzopoulou, Christos Liatsos, Ioanna-Konstantina Sgantzou, Jürg Knuchel, Michael Doulberis

**Affiliations:** 1 Greece Second Medical Clinic, School of Medicine, Ippokration Hospital, Aristotle University of Thessaloniki, Macedonia – Greece; 2 Greece Department of Gastroenterology, University Hospital of Larisa, Larisa – Greece; 3 Greece First Laboratory of Pharmacology, School of Medicine, Aristotle University of Thessaloniki, Macedonia – Greece; 4 Greece Department of Nutritional Sciences and Dietetics, School of Health Sciences, International Hellenic University, Macedonia – Greece; 5 Greece School of Healthcare Sciences, Midwifery Department, University of West Macedonia, Macedonia – Greece; 6 Greecee Department of Gastroenterology - General Military Hospital of Athens, Athens - Greecee; 7 Greecee Radiology Department, University Hospital of Larisa, Larisa – Greecee; 8 Switzerland Division of Gastroenterology and Hepatology, Medical University Department, Aarau – Switzerland

**Keywords:** Helicobacter pylori, Hypertension, Metabolic Syndrome, Atherosclerosis, Dyslipidemias

**To the Editor,**

In their meta-analysis, Huang et al.^1^ concluded that *Helicobacter pylori* infection ( *H. pylori* infection) is positively associated with systemic arterial hypertension, particularly by introducing the diagnostic 13C-urea breath test, signifying current *H. pylori* infection.

In this regard, systemic arterial hypertension is one of the most significant parameters of the metabolic syndrome (MetS), and its pathogenesis may mostly comprise of a noxious interplay between vascular, renal, neural, and hormonal mechanisms, of which augmented activation of the sympathetic nervous system (SNS) and the renin-angiotensin-aldosterone system (RAAS) predominate.^2^ Augmented activity of SNS is a usual feature of resistant systemic arterial hypertension, accompanied by increased release of norepinephrine, signifying a neurogenic element that contributes to the development of systemic arterial hypertension; and overactivation of SNS is associated with morbidity and mortality of MetS-related cardiovascular disorders.^3^ Moreover, RAAS dysregulation, including the systemic and brain RAAS, has been documented as one of the chief causes of several types of systemic arterial hypertension; and RAAS overactivation also contributes to MetS-associated obesity and cardiovascular morbidity and mortality.^4^

Likewise, *H. pylori* infection is also associated with MetS-related systemic pathologies, especially cardio-cerebrovascular and neurodegenerative diseases, the endpoints of MetS.^5 - 8^ Specifically, *H. pylori* infection seems to contribute to insulin resistance (IR), the chief underlying mechanism responsible for MetS,^9^ which also plays n critical role in the pathogenesis and progression of systemic arterial hypertension-triggered target organ injuries.^10^ MetS contributes to an increased risk of developing atherosclerosis,^11^ and in this respect, invasion of *H. pylori* into atheroma has been detected by introducing polymerase chain reaction (PCR).^12^ Direct *H. pylori* colonization in the arterial walls has been observed. *H. pylori* is associated with arterial stiffness, an early marker of systemic atherosclerosis correlated with systemic arterial hypertension and an independent predictor of cardiovascular complications and all-cause mortality. Thus, *H. pylori* have been associated with MetS-related atherosclerosis via a diversity of involved mechanisms, thereby potentially triggering systemic arterial hypertension. *H. pylori* infection might independently be involved in atherosclerosis and arterial hypertension through mechanisms distinct from the conventional causes of atherosclerosis, including the three non-conventional coronary artery disease risk factors homocysteine, fibrinogen and lipoprotein(a).^6 , 13 , 14^

Besides, MetS-related dyslipidemia is linked with systemic arterial hypertension^15^ and in this regard, chronic *H. pylori* infection can trigger abnormal lipid metabolism of the host, including, beyond the mentioned lipoprotein(a), low-density lipoprotein cholesterol (LDL-C), high-density lipoprotein cholesterol (HDL-C), and total cholesterol (TC),^16^ also mentioned by the authors;^1^ *H. pylori* -related lower HDL-C appears to promote dyslipidemia.^17^ In contrast, *H. pylori* eradication significantly decreases the levels of TC, TG, LDL-C and fibrinogen, an independent risk factor for cardiovascular disease,^6^ whereas increases HDL-C concentrations.^18 , 19^ Moreover, beyond dyslipidemia and systemic arterial hypertension, *H. pylori* eradication also improves other MetS-related parameters, including body mass index (BMI),^20^ IR,^21^ and total oxidant status.^22^ Therefore, eradication *of H. pylori* infection reduces the occurrence of MetS-related dyslipidemia and other parameters including systemic arterial hypertension,^23 , 24^ thereby potentially preventing the occurrence of MetS-related cardiovascular disease accompanied by arterial hypertension.

Recent data indicate that MetS-related sarcopenia, *H. pylori* infection, dyslipidemia, systemic arterial hypertension, diabetes mellitus, smoking, alcohol consumption, and diet (salty and/or spicy diets) are linked with precancerous gastric mucosa lesions, including gastric atrophy, intestinal metaplasia, and dysplasia.^25^ In this respect, interesting recent evidence also indicates that bariatric patients with *H. pylori* infection display baseline significantly high rates of the mentioned gastric pre-malignant lesions, including gastric atrophy and intestinal metaplasia accompanied with IR and arterial hypertension.^26^

Finally, nonalcoholic fatty liver disease (NAFLD), recently renamed as metabolic dysfunction-associated fatty liver disease (MAFLD), is the hepatic component of MetS also associated with *H. pylori* infection, which appears to contribute to its development and progression;^27^ NAFLD/MAFLD is associated with an about 1.6-fold augmented risk of developing systemic arterial hypertension; and MetS- related characteristics including high BMI, dyslipidemia, and type 2 diabetes mellitus in the setting of *H. pylori* infection exhibit a greater tendency for the development of NAFLD/MAFLD. In this regard, recent data indicate that *H. pylori* infection is connected with IR and augmented intestinal permeability, which could contribute to the development of NAFLD/MAFLD;^28^ and active *H. pylori* infection is independently positively associated with the severity of nonalcoholic steatohepatitis and fibrosis, findings suggesting probable clinical implications.^27^ Among patients with NAFLD/MAFLD, the prevalence of arterial hypertension varies from 40-70%, and relative studies have shown that NAFLD/MAFLD is strongly related to the augmented risk of systemic arterial prehypertension and hypertension.^29^ In contrast, beyond the reduction of the mentioned systemic arterial hypertension, *H. pylori* eradication particularly increases HDL-C and reduces LDL-C,^30^ thus restoring the cardioprotective activity of the HDL-C/LDL-C ratio and diminishing the cardiovascular risk linked to MAFLD.^30^

Viewing the aforementioned data, *H. pylori* infection seems to display pleiotropic effects beyond the gastrointestinal tract and rising evidence associates it with MetS, including systemic arterial hypertension. Further research is warranted, however, to clarify the potential impact of *H. pylori* related MetS on systemic arterial hypertension, which represents a serious public health problem with high global incidence and prevalence that continues to increase and may contribute to global high morbidity and mortality. Identifying *H. pylori* and MetS-related NAFLD/MAFLD and other relative disorders – as important risk factors for systemic arterial hypertension – may be helpful for improving the risk prediction, identifying primary preventive strategies, and selecting a therapeutic program for systemic arterial hypertension.
